# A Pragmatic Randomized Trial Comparing Suturing Techniques for Vesicourethral Anastomosis: One-Year Voiding Function Outcomes After Radical Prostatectomy

**DOI:** 10.3390/jcm14113934

**Published:** 2025-06-03

**Authors:** Utku Can, Erdinç Dinçer, Alper Coşkun, Mahmut Selman Mert, Cengiz Çanakçı, Cemal Göktaş

**Affiliations:** 1 Department of Urology, Health Sciences University, Kartal Dr Lutfi Kirdar City Hospital, 34865 Istanbul, Türkiye; drerdincdincer@gmail.com (E.D.); dr.alper05@gmail.com (A.C.); mselmanmert@gmail.com (M.S.M.); cengizcanakci@hotmail.com (C.Ç.); 2 Department of Urology, Avicenna Ataşehir Hospital, 34750 Istanbul, Türkiye; cemalgoktas@yahoo.com

**Keywords:** continue suture, interrupted suture, prostate cancer, radical prostatectomy, vesicourethral anastomosis

## Abstract

**Background:** Vesicourethral anastomosis (VUA) is a critical step in radical prostatectomy (RP), with interrupted suture (IS) and running suture (RS) as common techniques. However, there is no conclusive evidence suggesting the superiority of one technique over the other regarding voiding function. This study compares their effects on voiding function and continence recovery after retropubic RP. **Methods:** A two-group, parallel-design study included 70 patients with localized prostate cancer (pT1-pT2) undergoing retropubic RP by a single surgical team. Patients were randomized to VUA with IS (*n* = 35) or RS (*n* = 35). The primary outcomes included uroflowmetry parameters—maximum flow rate (MFR), voiding volume (VV)—post-void residual volume (PVR), urinary function assessed by the International Prostate Symptom Score (IPSS), and continence recovery. These outcomes were measured preoperatively and at 1, 3, 6, and 12 months post-surgery. Secondary outcomes included surgical parameters, perioperative complications and one-year oncological outcomes. **Results:** Suturing time was shorter for RS than IS (21 vs. 33 min, *p* = 0.001). Minimal anastomotic leakage occurred more frequently in the IS group (23% vs. 9%), while long-term anastomotic stenosis rates were comparable between RS and IS groups (12% vs. 9%). IS demonstrated significantly higher MFR at 1-month post-surgery (23.3 vs. 17.2 mL/s, *p* = 0.003). In subsequent follow-ups (3, 6, and 12 months), the mean MFR remained higher in the IS group, though without statistical significance. Logistic regression favored IS for early MFR outcomes (OR 4.16; 95% CI, 1.22–14.18; *p* = 0.023). Continence recovery and IPSS scores were similar between groups. **Conclusions:** Both techniques are effective and safe. RS reduces suturing time and leakage risk, while IS improves early postoperative MFR.

## 1. Introduction

Radical prostatectomy (RP) is a widely used curative treatment for prostate cancer (PCa). Various surgical approaches—perineal, transperitoneal, and extraperitoneal—including open, laparoscopic, and robotic methods, aim to optimize efficiency and minimize complications [1,2]. Currently, it is estimated that 80% of all prostatectomies in the United States are performed using robotic surgery [3]. Conversely, open radical prostatectomy (ORP) remains widely used in many developing countries—including Africa, Latin America, and Asia—where, as of 2019, only 6% of all Da Vinci Robotic Systems were installed [4].

Despite advances in surgical methods, perioperative complications remain a major challenge, with morbidity rates reaching 26% in high-volume centers [5]. Among the intraoperative steps, vesicourethral anastomosis (VUA) is a key determinant of early continence recovery and complications such as urinary leakage or anastomotic stricture [6]. VUA quality directly impacts the risk of vesicourethral anastomotic stenosis (VUAS), which occurs in 2.1–7.5% of cases [7,8].

Two primary suturing techniques for VUA have been widely adopted: the interrupted suture (IS) and the running suture (RS) methods. RS is most commonly used in minimally invasive approaches, particularly in robotic-assisted (RARP) and laparoscopic RP (LRP), and was initially described by van Velthoven et al. [9]. It is also increasingly adopted in ORP, particularly in Asian centers [10]. In contrast, IS remains the standard in many open surgical practices, owing to its precision and ease of tension control [11]

Both IS and RS techniques offer distinct advantages and limitations. RS is generally regarded as a faster method, associated with shorter catheterization times and lower urine extravasation rates compared to IS while maintaining similar continence outcomes and not increasing the risk of anastomotic strictures [12].

Previous studies evaluating vesicourethral anastomosis (VUA) have primarily assessed voiding function based on continence status and the presence or absence of overt VUAS without incorporating objective flow-based metrics that reflect the dynamic physiology of micturition [12]. Given the technical challenges of controlling suture tension under limited exposure in open surgery, a more detailed and quantitative assessment of voiding function appears warranted.

This randomized trial was designed to address this gap by evaluating not only continence recovery and the incidence of clinically confirmed VUAS but also incorporating serial assessments of maximum flow rate (MFR), voiding volume (VV), and post-void residual volume (PVR) as objective markers of voiding performance. We hypothesized that different anastomotic configurations—even when yielding similar stricture rates—may exert distinct effects on postoperative voiding dynamics. To our knowledge, this is one of the first randomized studies to compare VUA suture techniques in open radical prostatectomy using both conventional clinical endpoints and flow-based voiding parameters.

## 2. Materials and Methods

### 2.1. Study Population and Data Collection

**Figure 1:**
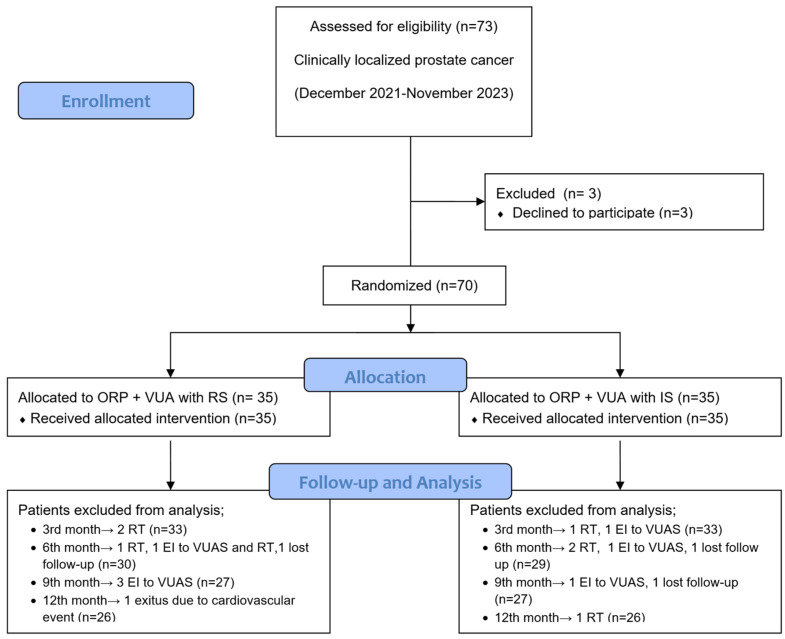
A CONSORT diagram is provided to illustrate the flow of participants throughout the study. ORP: open radical prostatectomy; RS: running suture; IS: interrupted suture; RT: radiotherapy; EI: endoscopic intervention; VUA: vesico-urethral anastomosis; VUAS: vesico-urethral anastomosis stricture.

A two-group, parallel-design pragmatic study was conducted involving 70 consecutive patients with clinically localized (pT1-pT2) PCa undergoing primary ORP by an experienced surgical team (UC, ED) at Kartal Dr. Lutfi Kirdar City Hospital, a tertiary care institution (See Figure 1). Men with a history of acute urinary retention and/or urethral stricture were excluded. The optimal sample size for this study was calculated to be 70 patients (35 patients in each arm) by the power calculation (α = 0.05, β = 0.10, power: 90%) based on the preliminary data of 47 patients’ MFR values in uroflowmetry at one month after surgery (17% better results in IS group). Participants were randomly assigned to the study groups using computer-generated random numbers in Microsoft Excel, ensuring allocation concealment. The surgical team was not informed of the treatment allocation, but the electronic medical record was updated with the surgery details as required by institutional regulations. Patient selection began after completing at least 40 cases for each anastomotic technique to avoid learning curve bias.

Participants provided written informed consent for their clinical data to be recorded. The study was approved by Kartal Dr. Lutfi Kirdar City Hospital Ethics Committee (registration no. 2021/514/214/32) and retrospectively registered on ClinicalTrials.gov (NCT06670924), with the date 1 November 2024. It adhered to the Declaration of Helsinki and Good Clinical Practice guidelines.

### 2.2. Surgical Techniques

Retropubic RP was performed according to Walsh’s technique with slight modifications [13]. Standardized procedures included posterior musculofascial reconstruction as described by Rocco et al. [14], a periurethral suspension stitch for anterior support, and bladder neck preservation. However, formal anterior reconstruction was not performed. Nerve-sparing procedures were performed when appropriate, and extended pelvic lymph node dissection was performed for high-risk patients, according to the Partin table.

The RS technique followed Van Velthovens’ method [9], widely used in LRP and RARP, with slight modifications. Two 3/0 absorbable monofilament (polydioxanone) sutures were used. The first needle was initiated from the bladder neck at 3 o’clock and terminated in the urethra at 9 o’clock. After completion of the posterior anastomosis, a transurethral catheter was placed. The second suture needle is passed from the bladder at 9 o’clock and ends in the urethra at 3 o’clock. The bladder neck and urethra are merged by gentle traction of the anterior and posterior sutures at 3 and 9 o’clock. The technique described by Walsh [13] for interrupted anastomotic suturing was applied with minor modifications. Six 3/0 absorbable monofilament (polydioxanone) sutures were placed at 1, 3, 5, 7, 9 and 11 o’clock to accomplish the VUA. The urethral catheter was removed under cystographic guidance on postoperative day 5; if leakage occurred, the procedure was repeated five days later.

### 2.3. Outcome Assessment

The study adhered to the CONSORT-PRO guidelines for patient-reported outcomes [15]. The primary outcomes included uroflowmetry parameters (MFR, VV), PVR as well as urinary function assessed by International Prostate Symptom Score (IPSS), and urinary function-related bother by IPSS quality of life question, with responses ranging from 0 to 6. Urinary continence recovery within one year of RP was defined as patient-reported use of zero pads or one security pad per day. These outcomes were measured preoperatively and at 1, 3, 6, and 12 months post-surgery. Secondary outcomes included surgical parameters, perioperative complications assessed using the Clavien–Dindo scoring system [16], and one-year oncological outcomes. Vesicourethral anastomotic stenosis (VUAS) was evaluated in patients presenting with obstructive signs, including persistently reduced MFR on serial uroflowmetry or voiding symptoms suggestive of bladder outlet obstruction. Diagnostic urethroscopy was performed under sedoanalgesia. VUAS was defined as narrowing at the VUA that prevented passage of a 21 Fr rigid urethrotome. Confirmed cases were treated with endoscopic dilation or direct visual internal urethrotomy (DVIU) and included in the final analysis. Biochemical recurrence was defined as a postoperative PSA > 0.2 ng/mL. Patients with suspected local recurrence were referred for salvage radiotherapy. Those receiving radiotherapy or endoscopic treatment for VUAS were excluded from subsequent follow-up evaluations to avoid confounding functional outcomes (see Figure 1).

Data were securely collected by an independent administrator in a password-protected database, ensuring confidentiality and eliminating bias from surgical or postoperative care teams.

### 2.4. Statistical Analysis

Statistical analyses were performed using SPSS software (version 22.0; IBM Corp., Armonk, NY, USA). Descriptive statistics were used to summarize baseline characteristics. Categorical variables were compared using Pearson’s chi-square or Fisher’s exact tests, and continuous variables were analyzed with the Mann–Whitney U test due to non-normal distribution. Bonferroni correction was applied for multiple timepoint comparisons of urinary incontinence outcomes. Logistic regression analysis was conducted to assess the association between suture technique and early postoperative voiding function, adjusting for potential confounders. The results of this analysis were visually presented using a forest plot. A *p*-value < 0.05 was considered statistically significant.

## 3. Results

### 3.1. Baseline Demographics

Table 1 summarizes patient characteristics and perioperative data. The mean age was 63.4 ± 6.9 years in the RS group and 65.3 ± 6.5 years in the IS group (*p* = 0.218). No significant differences were observed in initial PSA levels, body mass index (BMI), or Charlson Comorbidity Index (CCI) scores between the groups. The distribution of biopsy ISUP grades and the number of positive cores were comparable. Risk stratification by D’Amico classification was also similar, with intermediate and high-risk patients evenly distributed.

### 3.2. Perioperative Findings, Oncological Results and Complications

The RS group demonstrated significantly shorter suturing and operative times compared to the IS group (21 vs. 33 min, *p* = 0.001; 167 vs. 187 min, *p* = 0.003). Blood loss, drainage volume, catheterization time, and hospital stay were similar between the two groups.

Pathological analysis revealed a statistically significant difference in tumor staging between groups. Organ-confined disease (pT2) was more common in the RS group compared to the IS group (71% vs. 46%, *p* = 0.020), whereas extracapsular extension (pT3a) was notably higher in the IS group (51% vs. 14%). Seminal vesicle invasion (pT3b) was infrequent in both groups but slightly more observed in the RS group (14% vs. 3%).

Positive surgical margins (PSMs) were detected in 26% of RS patients and 40% of IS patients, a difference that did not reach statistical significance (*p* = 0.203). Although higher PSM rates were observed in the IS group, these did not translate into worse short-term oncological outcomes. At 12-month follow-up, biochemical recurrence (BCR)-free survival was comparable between the groups: 89% in the RS group and 91% in the IS group (*p* = 0.690). Four patients in each group received either salvage or adjuvant radiotherapy during follow-up, indicating similar rates of oncological treatment escalation. Despite the differences in pathological stage and PSM rates, no significant disparity in short-term oncological control was observed between the groups.

Table 2 presents complications classified by the Clavien–Dindo system. Minimal anastomotic leakage on postoperative day 5 was observed in three RS patients and eight IS patients (*p* = 0.101). By day 10, catheterization was successfully terminated in all patients. Two RS patients developed acute urinary retention post-catheterization, which resolved with prolonged catheterization and anti-inflammatory treatment. Anastomotic stenosis occurred in four RS patients and three IS patients during the one-year follow-up and was treated with an endoscopic bladder neck incision. No significant differences in complication rates were found between the groups (*p* = 0.808).

### 3.3. Continence Recovery, Uroflowmetry Parameters and International Prostate Symptom Scores

**Figure 2:**
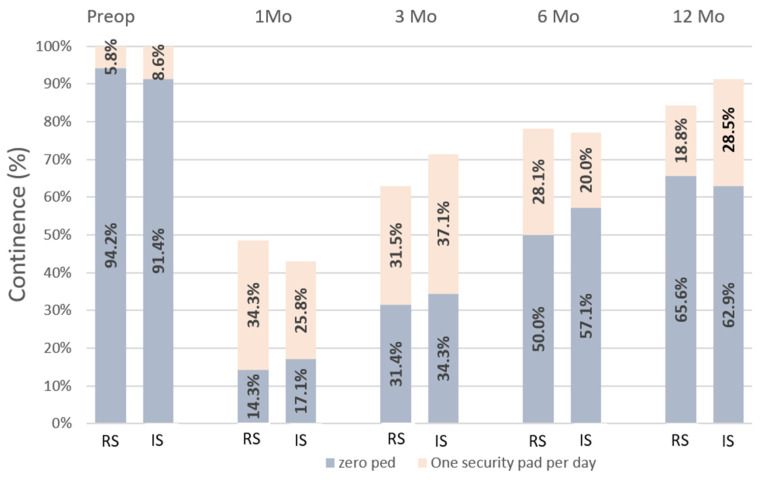
Comparison of continence status before surgery and 1, 3, 6 and 12. months after catheter removal in patients undergoing ORP with running or interrupted vesico-urethral anastomosis. RS = running suture; IS = interrupted suture; ORP = open radical prostatectomy.

All parameters were compared preoperatively and at 1, 3, 6, and 12 months post-surgery (Figure 2). At 4 weeks, 49% of RS patients and 43% of IS patients were continent (*p* = 0.631), with pad-free rates of 14% and 17%, respectively (*p* = 0.743). At 12 months, continence rates were 84% for RS and 91% for IS (*p* = 0.374), with pad-free rates of 66% and 63% (*p* = 0.813).

**Figure 3:**
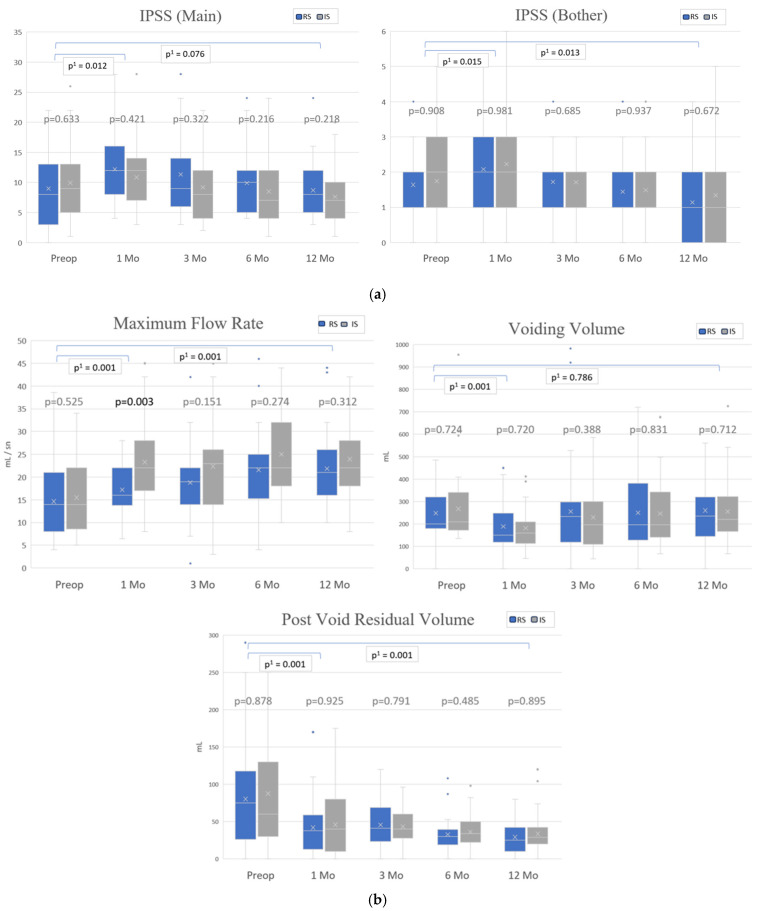
(**a**) Box plots of IPSS urinary (main) and bother scores at different time points by groups. (**b**) Box plots of voiding parameters at different time points by groups. RS = running suture; IS = interrupted suture. *p*: Comparison between RS and IS; *p*^1^: Time-based changes in overall cohort. *p* < 0.05 considered significant.

The IPSS urinary function and bother scores were not significantly different between the two cohorts at any time point (Figure 3a). Analysis of all patients showed that scores increased significantly compared to preoperative values at month 1 (9.5 vs. 11.5, *p*^1^ = 0.012 and 1.7 vs. 2.2, *p*^1^ = 0.015) and decreased below preoperative values at month 12 (9.5 vs. 8.2, *p*^1^ = 0.076 and 1.7 vs. 1.3, *p*^1^ = 0.013).

**Figure 4:**
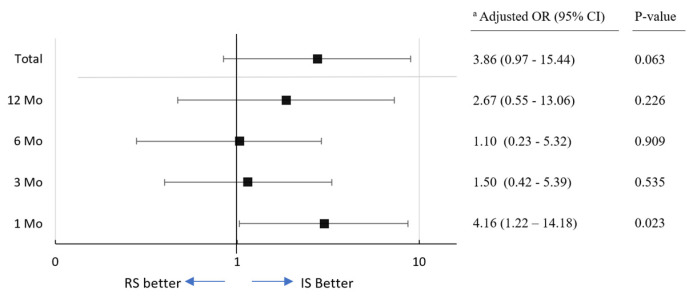
Forest plot for the MFR at overall and each timepoint, stratified by the preoperative median value. The interrupted suture group had a significantly better outcome at the one-month visit when compared with the RS group. ^a^ Adjusted for age, body mass index, Charlson comorbidity index score, continence status and voiding volume at related month. RS = running suture, IS = interrupted suture, OR = odds ratio, CI = confidence interval.

Box plots for MFR, VV, and PVR are shown in Figure 3b. The mean MFR values were significantly lower in the RS group (*n* = 35) compared to the IS group (*n* = 35) at 1-month post-surgery (17.2 vs. 23.3; *p* = 0.003). Similarly, among patients with a voiding volume ≥ 150 cc at the same visit, the mean MFR was significantly lower in the RS group (*n* = 19) than in the IS group (*n* = 21) (18.6 vs. 26.1; *p* = 0.008). At later follow-ups (3, 6, and 12 months), mean MFR values remained higher in the IS group, though differences were not statistically significant. Patients with MFR ≥ 15 mL/s were labeled as having a “strong voiding pattern”, while those <15 mL/s were classified as having a “weak voiding pattern”. Logistic regression analysis was performed to show the relationship between the voiding pattern and suture types following adjustment for potential confounding variables, including age, BMI, CCI, presence of incontinence and median VV. A forest plot (Figure 4) showed that the IS group had significantly better outcomes at 1 month when compared with the RS group [Odds Ratio (OR) 4.16 (1.22–14.18); 95% confidence interval (CI), *p* = 0.023].

## 4. Discussion

The most notable outcome of this comparative study is the difference in the time required for each technique. A meta-analysis demonstrated that VUA performed with RS is significantly faster than with IS in both overall and subgroup analyses (LRP and ORP) [12]. This finding aligns with prior research in other surgical fields, such as microvascular anastomosis, where RS has also been shown to be more time-efficient than IS [17]. A shorter operative time can have important clinical implications, as it is associated with a lower risk of intraoperative and postoperative complications [18]. Consistent with these findings, our study indicated that RS positively impacted both anastomosis time and total operative time.

The length of hospitalization was primarily determined by the recovery of bowel movements and the reduction in drainage volume. However, comparing hospitalization durations with similar studies is challenging due to differences in clinical practices and health insurance systems across countries [19]. In our study, the median hospital stay was 4 days for both groups, aligning with the reported median lengths of stay for LRP or RARP, which range from 3.5 to 8.8 days for IS and 3.1 to 9.8 days for RS [12].

Cystography was typically performed between the fifth and seventh postoperative days. In ORP, particularly with the RS technique, watertightness rates exceeding 95% have been reported even with catheter removal as early as the third postoperative day [20]. In our study, catheters were removed on the fifth postoperative day if no leakage was observed, with repeat cystography performed if necessary. Although most cases of urinary extravasation are clinically insignificant, they can delay catheter removal, reduce patient quality of life, prolong hospitalization, increase healthcare costs, and elevate the risk of anastomotic stenosis [21]. A meta-analysis reported that extravasation was more frequent in IS groups, with an odds ratio of 2.36 [12]. Similarly, our study found a 14% lower extravasation rate with the RS technique, although the median catheterization time was comparable between groups (5 days).

Anastomotic stricture rates have generally been reported as comparable between interrupted and running suture techniques. Teber et al. reported similar rates (7/200 RS vs. 6/200 IS) [22], and Massoud and Lee observed near-identical incidences (1 in 50 and 1 in 47, respectively) [23,24]. In our study, long-term VUAS was observed in 12% of RS and 9% of IS cases. Although the difference was not statistically significant, the overall rate appears slightly higher than those in previous reports. For instance, a recent national database study by Britton et al. found a VUAS rate of only 4.8% among over 18,000 men [25]. This discrepancy may be due to differences in diagnostic thresholds: Britton et al. defined VUAS as failure to pass a 17 Fr endoscope, whereas our criterion was stricter—failure to pass a 21 Fr rigid urethrotome during endoscopic evaluation. Furthermore, our use of routine serial uroflowmetry may have enabled earlier or more frequent detection, including milder or subclinical cases. A consistent decline in MFR and altered voiding pattern were the main triggers for endoscopic investigation. While our findings do not warrant a firm recommendation for universal flowmetric screening, they suggest that serial uroflowmetry may enhance diagnostic sensitivity. Further prospective studies are needed to determine whether routine flow-based monitoring improves detection and outcomes in patients at risk for VUAS.

According to a 2012 meta-analysis in European Urology, 12-month urinary continence recovery was higher with RARP than with ORP (91% vs. 84%) [26]. In these studies, the RS technique was predominantly used for VUA in RARP cases, whereas the IS technique was utilized in ORP cases. Matsuyama et al. [27] hypothesized that differences in continence outcomes could be attributed to variations in anastomosis techniques. Their 2015 randomized study comparing suture techniques in ORP found higher 1-month continence rates with RS, supporting this hypothesis. In contrast, an extensive randomized trial published in The Lancet in 2018 by Coughlin et al. [28] reported similar urinary continence rates for ORP (VUA with IS) and RARP (VUA with RS) at 6, 12, and 24 months. Furthermore, a meta-analysis of nine studies comparing anastomosis techniques found no significant differences in incontinence rates at 3, 6, and 12 months [12]. Consistent with these findings, our study showed no significant difference in continence recovery rates between the suture techniques at 1, 3, 6, and 12 months.

Both groups demonstrated significant improvements in MFR and reductions in post-void residual volume (PVR) over the 12-month follow-up period. The transient decrease in voided volume (VV) observed in the early postoperative period, likely attributable to post-prostatectomy incontinence, returned to preoperative levels over time. Master et al. conducted a comprehensive analysis of the voiding patterns and urinary symptoms in 125 patients who underwent RRP. Their findings align with our study, showing a progressive increase in MFR at postoperative months 2, 6, 14, and 20 compared to preoperative values (median 16.8, 20, 21, and 24 vs. 11.6 mL/s, respectively) [29].

When comparing the two groups, a notable observation was the superior MFR achieved with the IS technique compared to the RS technique at similar VV and PVR levels. It has been suggested that a tension-free anastomosis should be employed during RS in ORP [10]. However, achieving the appropriate tension, particularly in open surgery, can be technically challenging. It is hypothesized that the RS technique may restrict the relaxation capacity of the urethra-bladder neck complex, potentially leading to functional stenosis in the early postoperative period. While this may not result in clinically significant stenosis requiring intervention, it could negatively impact patients’ voiding flow rates. This hypothesis may not be applicable to the Velthoven continuous anastomosis technique used in LRP and RARP. In these approaches, the tissue apposition during VUA is achieved incrementally under direct visualization, allowing real-time adjustment of suture tension. In contrast, open surgery involves finalizing the anastomosis at the last knot, and although this step is described in the literature as requiring “gentle traction,” standardizing optimal suture tension remains challenging. The force applied during tissue approximation varies depending on tissue density and thickness. Furthermore, differences in tissue oxygenation and blood flow between RS and IS techniques may exist. Animal studies have demonstrated reduced anastomotic blood flow and perianastomotic oxygenation with continuous sutures, potentially leading to impaired healing and higher complication rates [30]. In our study, part of the MFR reduction in both groups was attributable to patients with clinically confirmed anastomotic strictures requiring intervention. These cases likely result from a multifactorial process involving patient characteristics, surgical technique, and healing-related complications that promote fibrosis and stricture formation [25]. However, consistent with prior meta-analyses, we found no significant difference in VUAS rates between the suture techniques [12]. Notably, despite similar stricture incidence, mean MFR at 1 month was significantly lower in the RS group (17.2 vs. 23.3; *p* = 0.003). This suggests that reduced voiding flow may result not only from overt VUAS but also from subtle functional differences detectable through uroflowmetry. Incorporating flow-based assessments into postoperative evaluation may thus offer a more detailed understanding of early functional outcomes related to suture technique.

Despite observed differences in MFR, no significant difference was identified between the groups in terms of IPSS at any follow-up visit. The IPSS is a well-established tool for assessing lower urinary tract symptoms (LUTS) following radical prostatectomy (RP) [31]; however, it has not been shown to correlate strongly with bladder outlet obstruction or MFR [32]. In a randomized study comparing anastomosis techniques in ORP, the RS technique was associated with superior Expanded Prostate Cancer Index Composite (EPIC) urinary function and quality of life (QoL) scores at the 1-month follow-up. However, outcomes were comparable between the techniques in subsequent months [24]. Interestingly, the study also reported higher continence rates in the RS group during the first postoperative month, which may have contributed to the improved urinary scores. In contrast, our findings indicated no significant differences in IPSS or continence recovery rates between the two suture techniques. Across all patients, IPSS urinary and bother scores increased in the early postoperative period but decreased below preoperative levels by the end of the first year. Walker et al. [32] conducted preoperative urodynamic evaluations and suggested that the transient worsening in IPSS observed in the early postoperative period could be attributed to de novo detrusor overactivity and urodynamic filling phase abnormalities. Similarly, Prabhu et al. [33] conducted a 10-year follow-up study evaluating the long-term effects of RP on LUTS. Their results demonstrated that RP effectively improved LUTS and prevented age-related progression of these symptoms over the long term. However, while the increase in MFR observed in some patients following RP may positively influence LUTS, post-prostatectomy incontinence can have a counterbalancing negative impact. We posit that multiple factors contribute to variations in IPSS, underscoring the complexity of its interpretation.

In our study, we utilized the classical Van Velthoven method for RS and a well-established Walsch IS technique commonly applied in open prostatectomy. Beyond suture type, variations in suture number, knot configuration, and placement may influence outcomes. Flammia et al. reported excellent early continence using a single-knot RS with posterior reconstruction [34]. Similarly, IS techniques have been optimized by altering suture number and position; some studies suggest that placing interrupted stitches at 3 and 9 o’clock positions may reduce sphincter trauma and improve early continence [35]. While barbed sutures and hybrid approaches have gained popularity in minimally invasive surgery, their isolated effect on postoperative voiding function remains unclear [36]. Taken together, it appears that suture technique, beyond RS or IS, must be considered as a multifactorial construct, in which tension distribution, anatomical alignment, and tissue handling may play equally decisive roles in early functional recovery.

This study offers several contributions to the existing literature. Unlike prior trials that primarily assessed continence recovery, our trial evaluated voiding function using both subjective (IPSS) and objective (uroflowmetry) tools. The early postoperative advantage observed in the interrupted suture group may be clinically relevant, particularly for patients with marginal baseline flow rates. Moreover, the findings underscore a technical limitation of the running suture method in open surgery, where fine-tuning suture tension is less controlled compared to minimally invasive approaches. Given that running sutures are widely used in LRP and RARP, our results may also have implications for early functional outcomes in those settings. To our knowledge, no prior randomized study has addressed this question using objective flow-based parameters, and our findings may serve as a reference for future research in minimally invasive platforms.

This study has several limitations. First, it was conducted by a single surgical team at an academic institution, which may limit the generalizability of the findings to other clinical settings or surgical teams. Second, although a preliminary study was performed, the surgical team had greater expertise in the interrupted suture technique, which may have influenced the outcomes. Third, the uroflowmetric values analyzed in the study represent instantaneous voiding flow and provide an estimation of obstruction severity related to VUA stenosis. However, these values are not definitive diagnostic tools. It is essential to acknowledge that a low MFR can also result from non-obstructive causes, such as an underactive bladder or the presence of incontinence. Additionally, although the groups were similar in baseline clinical parameters, paradoxically higher rates of pT3a disease and positive surgical margins were observed in the IS group. This may be partly explained by postoperative pathological upstaging and group imbalance due to limited sample size—both of which are recognized limitations in pragmatic clinical trials. These limitations underscore the need for validation through studies involving larger cohorts and multiple centers to enhance the robustness, external validity, and applicability of the findings.

## 5. Conclusions

Both approaches demonstrated favorable functional outcomes with acceptable complication rates. Although the RS technique appears to offer advantages such as shorter anastomotic time and reduced intraoperative leakage, most surgeons are likely to continue using the IS technique in ORP. This preference is driven by the superior postoperative MFR associated with IS, longstanding clinical practice patterns, and its status as a technically well-established method.

## Figures and Tables

**Table 1:** Relevant demographic, clinical and pathological variables for patients undergoing open radical prostatectomy (ORP) with running versus interrupted anastomosis suture.

		Running	Interrupted	*p* Value
No.pts		35	35	
Demographics and clinical findings				
	Age (yr), mean (SD)	63.4 ± 6.9	65.3 ± 6.5	0.218
	BMI (kg/m^2^), mean (SD)	26.3 ± 3.7	26 ± 3	0.538
	PSA (ng/dL), median (IQR)	8.2 (11.6)	7.8 (5.1)	0.378
	CCI score, *n* (%)			0.780
	≤3	27 (77)	26 (74)	
	>3	8 (23)	9 (26)	
	Biopsy ISUP, *n* (%)			1
	≤2	29 (83)	29 (83)	
	>2	6 (17)	6 (17)	
	Number of positive biopsy cores, *n* (%)			0.626
	≤3	20 (57)	22 (63)	
	>3	15 (43)	13 (37)	
	D’amico risk classification, *n* (%)			0.328
	Low	15 (43)	11 (31)	
	Intermediate	16 (46)	22 (6)	
	High	4 (11)	2 (6)	
Intraoperative findings				
	Amount of bleeding (cc), mean (SD)	664 ± 316	631 ± 267	0.731
	Operative time (min), mean (SD)	167 ± 28	187 ± 35	0.003
	Anastomosis time (min), median (IQR)	21 (6)	33 (8)	0.001
	PLND, *n* (%)	24 (69)	27 (77)	0.420
	Nerve-sparing surgery, *n*			0.513
	None	7	11	
	Unilateral	8	8	
	Bilateral	20	16	
Postoperative findings				
	Total amount of drainage (mL), median (IQR)	300 (140)	205 (561)	0.106
	Hospital stay (days), median (IQR)	4 (1)	4 (2)	0.345
	Catheterization time (days), median (IQR)	5 (1)	5 (5)	0.860
	No extravasation on the first cystogram, *n* (%)	32 (91)	27 (77)	0.101
Total follow-up period (months), median (IQR)		16 (8)	17 (8)	0.946
Pathological results and BCR				
	Prostate specimen weight (g), mean (SD)	47 ± 19	58 ± 20	0.463
	Pathological stage, *n* (%)			0.020
	T2	25 (71)	16 (46)	
	T3a	5 (14)	18 (51)	
	T3b	5 (14)	1 (3)	
	Positive surgical margin, *n* (%)	9 (26)	14 (40)	0.203
	No.pts with one-year biochemical recurrence-free, *n* (%)	31 (89)	32 (91)	0.690
	No.pts receiving radiotherapy (Salvage or adjuvant), *n* (%)	4 (11)	4 (11)	1

**Table 2:** Intraoperative and postoperative complications (Clavien–Dindo classification).

		Running *n* = 35	Interrupted *n* = 35	*p* Value	Intervention
Clavien grade/Complication Intraoperative					
II	Bleeding	6 (17%)	4 (12%)	0.721	Blood Transfusion
Postoperative short period (≤6 weeks)					
I	Wound infection	2 (6%)	2 (6%)	1	Antibiotherapy, bedside intervention
	Prolonged drainage	0 (0%)	1 (3%)	0.314	Long-time urethral catheterization
	Acute urinary retention	2 (6%)	0 (0%)	0.151	Urethral catheterization
Id	Anastomotic leakage on the first cystourethrogram	3 (9%)	8 (23%)	0.101	Long-time urethral catheterization
II	Urinary infection	2 (6%)	2 (6%)	1	Antibiotherapy
	Hematuria	4 (12%)	5 (14%)	0.721	Conservative approach
	Lymphorrhoea	1 (3%)	0 (0%)	0.314	Suction drainage
Postoperative long period (>6 weeks)					
IIIa	Anastomotic stricture	4 (12%)	3 (9%)	0.690	Endoscopic bladder neck incision
Total (At least one complication)		21 (60%)	20 (57%)	0.808	

## Data Availability

The data that support the findings of this study are not publicly available due to their containing information that could compromise the privacy of research participants but are available from the corresponding author [UC, e-mail: utkucan99@yahoo.com] upon reasonable request.

## Abbreviations

RS = running suture; IS = interrupted suture; VUA = vesicourethral anastomosis; VUAS = vesicourethral anastomosis stenosis; Pca = prostate cancer; RP = radical prostatectomy; ORP = open radical prostatectomy; RARP = robotic-assisted radical prostatectomy; LRP = laparoscopic radical prostatectomy; MFR = maximum flow rate; VV = voiding volume; PVR = post-voiding residual volume; IPSS = International Prostate Symptom Score; BMI = body mass index; CCI = Charlson comorbidity index; PLND = pelvic lymph node dissection; RT = radiotherapy.
